# Atmospheric mercury inputs in montane soils increase with elevation: evidence from mercury isotope signatures

**DOI:** 10.1038/srep03322

**Published:** 2013-11-25

**Authors:** Hua Zhang, Run-sheng Yin, Xin-bin Feng, Jonas Sommar, Christopher W. N. Anderson, Atindra Sapkota, Xue-wu Fu, Thorjørn Larssen

**Affiliations:** 1 State Key Laboratory of Environmental Geochemistry, Institute of Geochemistry, Chinese Academy of Sciences, Guiyang, 550002, China; 2 Norwegian Institute for Water Research (NIVA), Gaustadalléen 21, 0349 Oslo, Norway; 3 State Key Laboratory of Ore Deposit Geochemistry, Institute of Geochemistry, Chinese Academy of Sciences, Guiyang, 550002, China; 4 Soil and Earth Sciences Group, Institute of Agriculture and Environment, Massey University, Palmerston North, New Zealand; 5 These authors contributed equally to this work.

## Abstract

The influence of topography on the biogeochemical cycle of mercury (Hg) has received relatively little attention. Here, we report the measurement of Hg species and their corresponding isotope composition in soil sampled along an elevational gradient transect on Mt. Leigong in subtropical southwestern China. The data are used to explain orography-related effects on the fate and behaviour of Hg species in montane environments. The total- and methyl-Hg concentrations in topsoil samples show a positive correlation with elevation. However, a negative elevation dependence was observed in the mass-dependent fractionation (MDF) and mass-independent fractionation (MIF) signatures of Hg isotopes. Both a MIF (Δ^199^Hg) binary mixing approach and the traditional inert element method indicate that the content of Hg derived from the atmosphere distinctly increases with altitude.

Mercury (Hg) is a persistent, semi-volatile element that can be ubiquitously detected throughout the world. Gaseous compounds dominate the pool of atmospheric Hg, with elemental Hg (Hg^0^) as the major constituent (>95%) and with gaseous oxidised compounds (GOM) and Hg bound to aerosols (Hg-p) as minor constituents^1^. Hg^0^ has a residence time in the atmosphere of 0.5 to 2 years^1^ and can be transported through the atmosphere far beyond the regions where it was emitted and thus deposited into pristine environments. Tremendous effort has been exerted in recent decades to understand the fate, transport and behaviour of Hg on both regional and global scales due to its potential adverse impacts on the health of humans and the environment^1^,^2^,^3^. In general, inorganic species predominate in the biogeochemical cycle of Hg, but various abiotic- and biotic-mediated pathways convert small amounts of Hg into neurotoxic methylated Hg (MeHg) species. Such organomercurials can effectively be bio-accumulated through aquatic food webs and even in some terrestrial plants (e.g., rice^3^), eventually posing a serious threat to humans through the consumption of fish and/or rice^2^.

Atmospheric Hg deposition is dominated largely by the physical scavenging of GOM and Hg-p^1^. Hg^0^ is known to exhibit bi-directional flux patterns and is associated with deposition velocities in the range of up to only a few mm s^−1^ (ref 4). In sharp contrast to Hg^0^, inorganic GOM species have intermediate vapour pressures (log_10_*p* (Pa) < 2) and partition favourably (i.e., Henry's law coefficient of ≥10^4^ M atm^−1^) to the aqueous phase. The dominant global sink for atmospheric Hg^0^ is gas-phase oxidation yielding GOM species, with most of the oxidation occurring in the middle and upper troposphere^5^. This tropospheric pool of GOM may, through subsidence and other down flow processes, be an important source of Hg^2+^ deposition in high-altitude surface sites^6^. For example, recent studies have provided evidence of a free tropospheric source of Hg in wet deposition to the western United States^7^,^8^.

Alpine regions are generally considered to be vulnerable ecological environments because of their weak capabilities for self-purification and self-recovery. Previous studies have shown that environments in these regions are critically sensitive to atmospheric Hg deposition, especially topsoil and vegetation, which are regarded as effective carriers of atmospheric Hg deposition^9^,^10^. Furthermore, alpine regions exhibit substantial differences in their climatic, biological and environmental characteristics with altitude, such as an increased atmospheric deposition due to high surface roughness as well as increased precipitation and cloud water interception and lower soil/foliage emissions due to low temperatures^11^,^12^. Mechanisms driven by specific orographic conditions may thus act together to cause mountainous areas to become convergence zones for Hg (the ‘mountain trapping effect’).

Nevertheless, few studies investigating Hg have been conducted in appropriate mountainous areas along well-defined elevational transects^9^,^10^,^13^. Consequently, in some respects, our knowledge of the biogeochemical cycling of Hg in mountainous ecosystems remains limited. In recent decades, there has been great progress in simulations of the global/hemispheric or regional Hg distribution and examinations of the source-receptor relationship using various modelling systems (e.g., CMAQ-Hg^14^ and GEOS-Chem^15^), although surprisingly little attention appears to have been given to orographic effects. Ignoring the possibility of a mountain trapping effect on Hg may, however, hamper the validity of modelling results. Broadly defined, 27% of the Earth's landmass can be classified as mountainous, including plateaus and hills, and is inhabited by 22% of the world's population^16^. In China, mountains account for two-thirds of the total land area, with >50% composed of mountains and plateaus with elevations > 1,000 m a.s.l^16^.. Therefore, studies supplying the missing Hg pollution data for the mountain ecosystems in China and worldwide are of particular importance for evaluating the roles of these areas in global Hg distribution and cycling.

Soil compartments have typically been used to determine atmospheric contaminant deposition because the soil is the major terrestrial repository of contaminants, reflecting decades to centuries of wet and dry deposition^8^,^17^. A recent study on areal Hg mass conducted in 14 forests across the U.S. has shown that soil is the biggest terrestrial repository for Hg (90%), followed by litter (8%) and aboveground biomass (<1%)^18^. However, quantifying the Hg sources in soil contaminant pools from atmospheric input remains challenging because mountain soil Hg concentrations stem from both local mineral composition, as a natural background, and atmospheric input of natural and anthropogenic origins^9^.

Of potential benefit to our understanding of Hg sources has been research, in the past decade, into the isotopic dimension of environmental Hg cycling of Hg. This research has proven to be a powerful approach in tracing the sources of Hg and quantifying the physicochemical processes that affect Hg cycling. Recent studies have demonstrated that Hg isotope ratios vary widely among different source materials and that Hg isotopes can be systematically fractionated during specific environmental processes^19^,^20^,^21^. In addition to mass-dependent fractionation (MDF), mass-independent fractionation (MIF) of the odd-mass Hg isotopes (^199^Hg and ^201^Hg) may occur as a consequence of mechanisms such as the magnetic isotope effect (MIE)^22^,^23^ and nuclear volume effect (NVE)^20^,^24^. A combined analysis of MDF and MIF Hg signatures in topsoil samples can be used as an effective tracer for atmospheric sources^25^.

Mt. Leigong (‘god of thunder’) is the highest peak (2,179 m a.s.l., 26.39°N, 108.20°E) within the Miaoling Range of Guizhou Province, China (Fig. S1) and was selected for the present study. The mountain, which is regarded as holy by local ethnic minorities, is located within a National Nature Reserve (473 km^2^). Because the southwestern slope is the only slope with a relatively consistent gradient along its length^11^, it was used for sample collection. The bedrock on Mt. Leigong is primarily composed of low-grade metamorphic rock from the Pre-Sinian Age, and the soil type is dominated by ultisol^26^. The relative proximity to the South China Sea (~750 km) causes Mt. Leigong to be greatly affected by the summer monsoon, resulting in abundant rainfall (annual precipitation ranges from ~1250 mm in the lowland to >1600 mm in the summit zone). Following an altitudinal gradient, the mean annual temperature decreases by 0.46°C per 100 m of elevation gain to a low of 9.2°C in the highest zone^12^. The summit is frequently shrouded in persistent fog (~300 days yr^−1^), and the piedmont experiences fog on <25% of the days in the year^11^,^26^.

The present study was conducted principally to test the hypothesis that mountainous regions act as regional convergence zones for atmospheric Hg, analogous to classes of organic persistent pollutants^27^. The survey of Hg in the topsoil along an elevational transect is linked to a seasonally resolved monitoring program conducted at the summit that examines Hg in the air and Hg deposition^10^. Hg isotope signatures and inert element tracers were used to identify and quantify the Hg sources and to explore the possible underlying mechanisms of ‘mountain trapping effect’ of Hg.

## Results

### Hg levels and distribution along the elevation gradient

**Figure 1:**
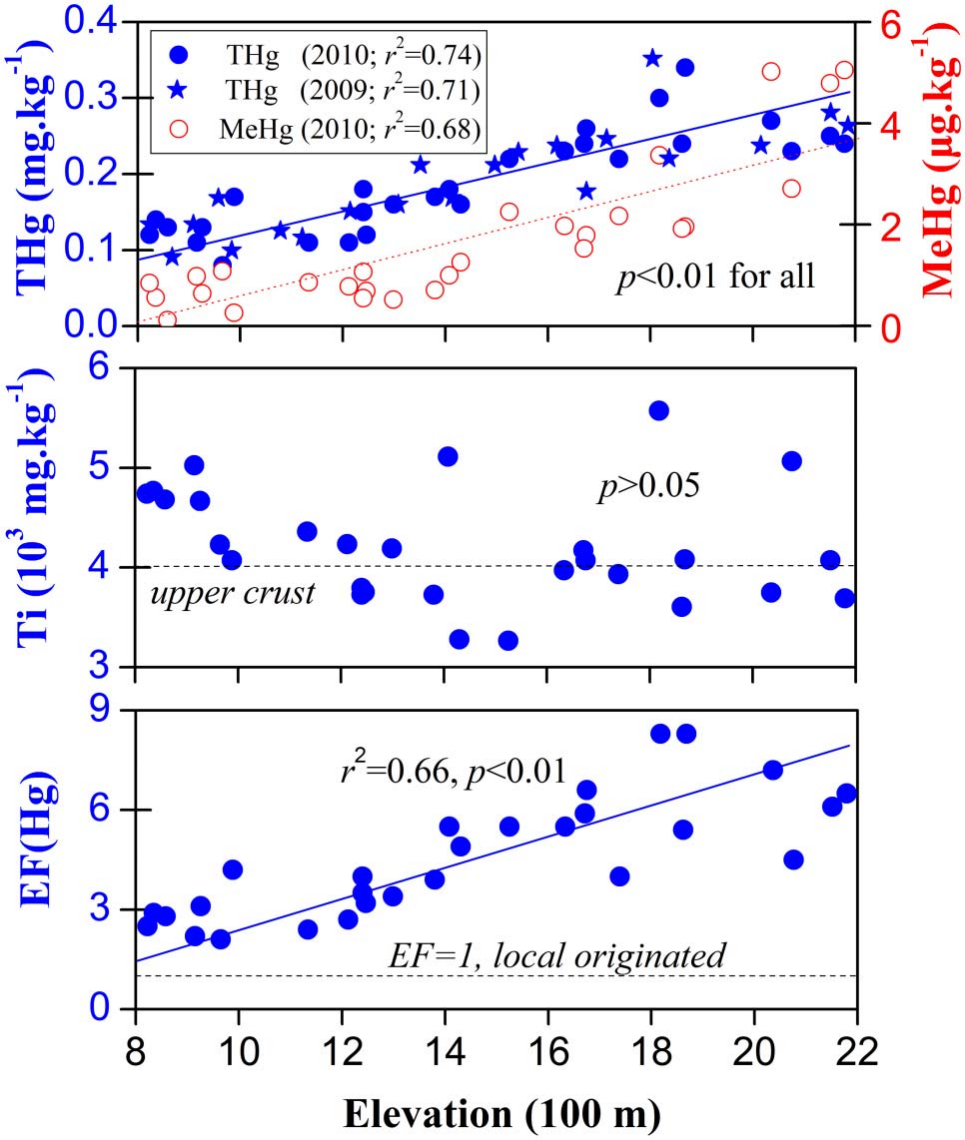
Scatter plots of soil THg and MeHg contents (upper panel), soil Ti content (centre panel) and calculated enrichment factors (EF(Hg) = (Hg/Ti)*_soil_*/(Hg/Ti)*_crust_*) (lower panel) versus elevation.

Elevated total Hg (THg) levels were observed in the topsoil samples compared with the gross background average of China (0.052 mg·kg^−1^ (ref 28)), with a mean (range) of 0.18 (0.07–0.34) mg·kg^−1^ and 0.20 (0.08–0.38) mg·kg^−1^ in samples collected in September in 2009 and 2010, respectively (Fig. 1). The mean (range) of the MeHg content in the corresponding samples for 2010 was 2.16 (0.26–5.05) μg.kg^−1^. No significant difference (ANOVA, *p* > 0.05) in the THg levels was observed between the samples collected at equal altitudes in 2009 and 2010; therefore, these data will be combined in the following discussion. The soil THg level (mean 0.19 mg·kg^−1^) for Mt. Leigong compares favourably with the distributions reported for forest soils from remote areas in the U.S. (0.15 mg·kg^−1^), Norway (0.19 mg·kg^−1^) and Sweden (0.25 mg·kg^−1^)^9^.

The THg and MeHg concentrations in the topsoil show an increasing trend with altitude (*r*^2^ = 0.68–0.71, *p* < 0.01 for both) (Fig. 1). The linear fits resulted in slopes of 0.12 μg.kg^−1^ m^−1^ and 3.1 ng.kg^−1^ m^−1^ for THg and MeHg, respectively. Both THg and MeHg concentrations in the soils near the summit increased to approximately three times higher than those in the piedmont areas. Furthermore, the THg concentration in the moss and litterfall samples exhibited similar trends with elevation (*r*^2^ = 0.33–0.39, *p* < 0.01 for both) (Fig. S2–S3). These results may indicate an altitudinal magnification effect (i.e., mountain trapping effect) of atmospheric Hg on Mt. Leigong. Several studies in high-altitude regions have revealed enhanced concentrations and deposition rates of Hg^10^,^17^,^18^. Hence, a ubiquitous phenomenon in which these regions function as regional convergence zones for atmospheric Hg may exist.

### Hg isotope ratios distribution along the elevation gradient

**Figure 2:**
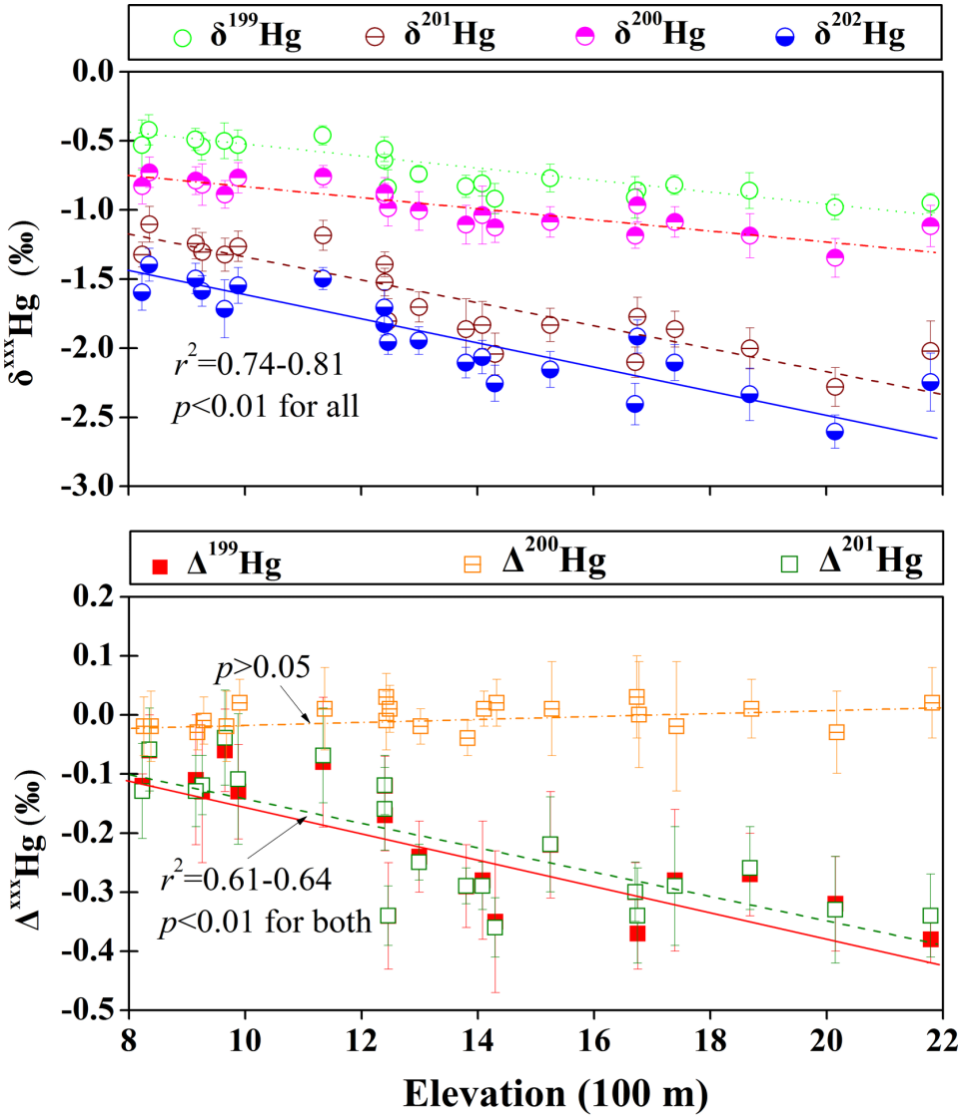
Scatter plots of mean δ^XXX^Hg (upper panel) and mean Δ^xxx^Hg (MIF, lower panel) isotope ratios in surface soil versus elevation. All error bars represent ± 2 s.d.

**Figure 3:**
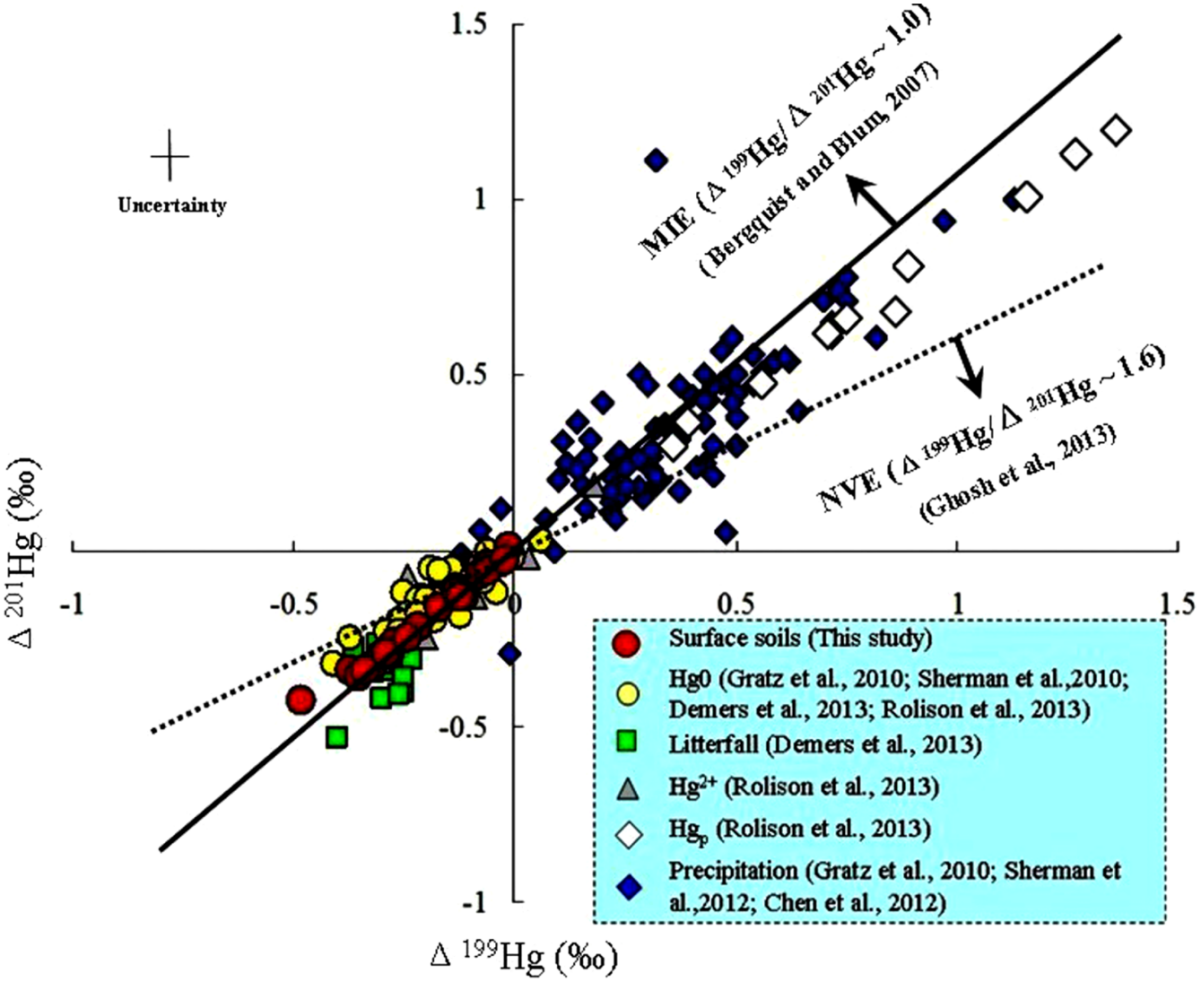
A comparison of the relationship between Δ^199^Hg and Δ^201^Hg from various studies (MIE = magnetic isotope effect; NVE = nuclear volume effect).

Significant MDF (within a 1.2‰ range for δ^202^Hg) and MIF (a 0.3‰ range for both Δ^201^Hg and Δ^199^Hg) signatures were observed in all soil samples (Fig. 2). A consistent negative trend with elevation was observed for the soil THg isotope ratio values (*r*^2^ = 0.61–0.82, *p* < 0.01) (Fig. 2), with the corresponding slopes (‰ per 100 m elevational gain) obtained by the linear fit of −0.039, −0.040, −0.083 and −0.083 for δ^xxx^Hg (xxx = 199, 200, 201 and 202, respectively) and of −0.020 and −0.021 for Δ^199^Hg and Δ^201^Hg, respectively. Furthermore, the Δ^201^Hg values were well correlated with the Δ^199^Hg values (*r*^2^ = 0.98, *p* < 0.01) (Fig. 3). No significant MIF of even isotopes (e.g., ^200^Hg and ^204^Hg) was observed in any of our investigated samples (lichen, soil and rock samples).

### Tracing and quantifying the atmospheric Hg inputs in soil samples

(1). Inert element method. The inert element method was employed to differentiate between Hg sources in the Mt. Leigong soil samples. Titanium (Ti), a conservative element in the chemical weathering process, was selected as the reference element to calculate the Hg enrichment factor (EF) (defined as EF[Hg]) based on the surface soil and upper crust concentrations^28^ according to the following equations: 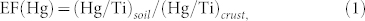

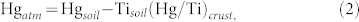


**Figure 4:**
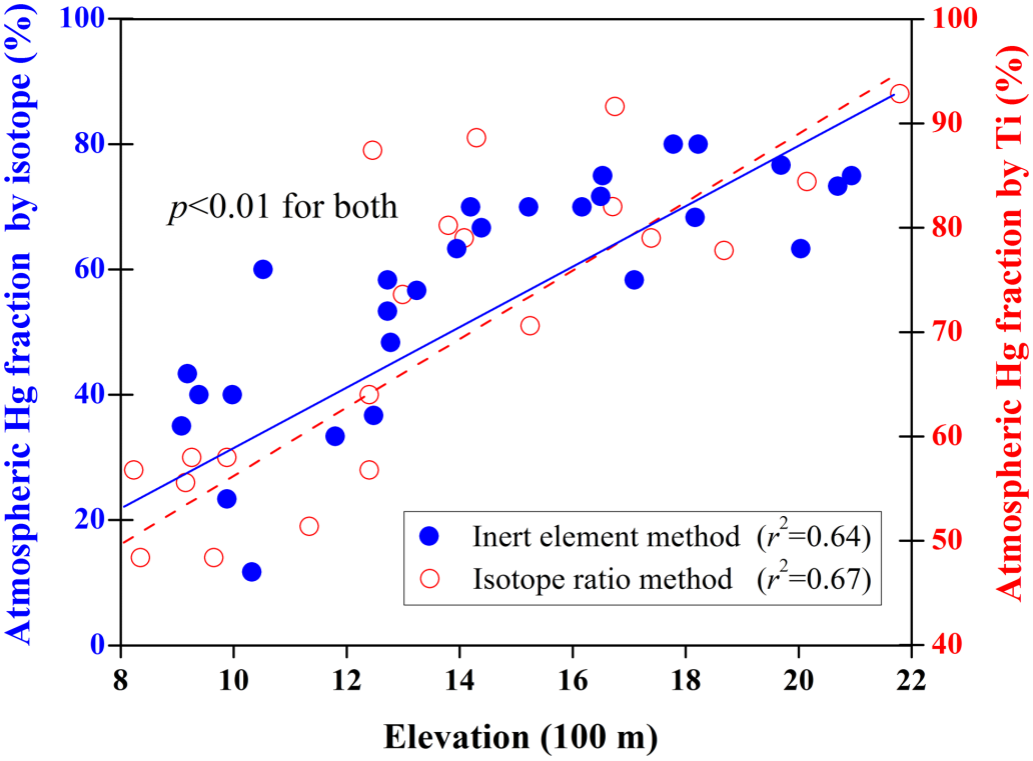
Predicted fractional contribution (%) of atmospheric input to the soil THg level as a function of elevation using the isotope ratio method (closed circles, left ordinate axis) and the inert element method (open circles, right ordinate axis).

As Fig. 1 illustrates, the EF(Hg) was positively correlated with elevation (*r*^2^ = 0.66, *p* < 0.01). An EF(Hg) value closer to 1 indicates that the Hg in the soil is of geogenic origin. The trend of increasing EF(Hg) values with increased elevation suggests that non-geogenic sources of Hg (assumed to be primarily Hg*_atm_*, or atmospheric input) increase with elevation. The ratio of atmospheric Hg to THg in the soil progressively decreased from approximately 90% at the summit to less than 50% at the base of the mountain (*r*^2^ = 0.64, *p* < 0.01) (Fig. 4), suggesting a significant contribution from non-geogenic sources (largely atmospheric input) to the surface soil Hg on Mt. Leigong, particularly at higher elevations.

(2). Hg isotope signature method. As suggested by Bergquist and Blum^19^, the application of stable Hg isotopes as a tool to discriminate the sources and transformations of Hg can be challenging, due to the fact that MDF signatures can result from numerous processes. Compared with MDF, MIF signatures are more specific to certain geochemical processes, and these might be identifiable by their Δ^199^Hg/Δ^201^Hg ratios. According to current knowledge, the MIF is expected to be unaltered by MDF processes, but can be changed by other MIF processes or through the mixing of Hg pools with different MIF signatures. Several studies have observed significant negative Δ^199^Hg values in epiphytic lichens used to identify atmospheric Hg deposition^29^,^30^,^31^. However, as shown in Fig. 3, recent studies on the direct analysis of atmospheric Hg species have showed positive Δ^199^Hg in particulate mercury (Hg_p_) andprecipitation, and slightly negative Δ^199^Hg in gaseous elemental Hg and its oxidised form (Hg^2+^)^25^,^32^,^33^,^34^,^35^. For example, Gratz et al^32^. collected precipitation and Hg^0^ samples in the Great Lakes region of the U.S. The precipitation samples showed a positive MIF (Δ^199^Hg: +0.04‰ to +0.52‰), whereas the Hg^0^ samples showed a negative MIF (Δ^199^Hg:−0.21‰ to +0.06‰). Sherman et al^25^. observed a slightly negative MIF (Δ^199^Hg: −0.11‰ to −0.22‰) in Hg^0^ collected from Arctic areas. Rolison et al^35^. investigated the isotopic composition of species-specific atmospheric Hg in a coastal environment of Grand Bay, Mississippi, U.S. According to their study, particulate Hg (Hg_p_) samples displayed a significant positive MIF (Δ^199^Hg: +0.36‰ to +1.36‰), reactive gaseous Hg^2+^ displayed an intermediate MIF (Δ^199^Hg: −0.28‰ to 0.18‰) and gaseous Hg^0^ (which contributed >95% of the total gaseous Hg) displayed a negative MIF (Δ^199^Hg: −0.41‰ to −0.03‰). These findings raise the question of the integrity of the Hg isotopes measured in lichens relative to atmospheric Hg, with the possibility of MIF during bioaccumulation. Terrestrial vegetation (including lichens) can accumulate Hg through the absorption of wet (e.g., precipitation) and dry (e.g., particulate Hg) atmospheric Hg deposition and through the incorporation of Hg^0^ through the stoma^31^. Atmospheric Hg can be fractionated during the process of incorporation by plants^31^,^34^, mosses and lichens^29^,^36^, with the lighter isotopes preferentially binding within the foliage. However, empirical evidence demonstrating a lack of MIF during metabolic processes has been shown in fish, microorganisms and terrestrial plants^31^,^37^,^38^. For example, Demers et al^34^. investigated the Hg isotopic composition in foliage samples of Aspen trees in a pristine forest in NE Wisconsin, U.S. They demonstrated an MDF of ~−3.0% in the δ^202^Hg values and a slight shift of ~−0.1% in the Δ^199^Hg values (perhaps due to the influence of the deposited Hg_p_) between the total atmospheric Hg pool (δ^202^Hg: −0.94‰ ± 0.35‰, 2 s.d., n = 12; Δ^199^Hg: −0.19‰ ± 0.04‰, 2 s.d., n = 12) and the foliage (δ^202^Hg: −2.14‰ ± 0.19‰, 2 s.d., n = 18; Δ^199^Hg: −0.30‰ ± 0.05‰, 2 s.d., n = 18). Yin et al^31^. measured the Hg isotopic composition in the rice foliage near Wanshan Mercury Mine in southwestern China. According to their study, an MDF of ~−3.0% in the δ^202^Hg value and insignificant MIF were observed between the Hg^0^ pool (δ^202^Hg: −2.15‰ ± 0.21‰, 2 s.d., n = 4; Δ^199^Hg: −0.29‰ ± 0.04‰, 2 s.d., n = 4) and the foliage (δ^202^Hg: −3.18‰ ± 0.21‰, 2 s.d., n = 6; Δ^199^Hg: −0.24‰ ± 0.08‰, 2 s.d., n = 6).

Litterfall is an important source of Hg in forest organic soils^17^,^18^. The uptake of isotopically lighter atmospheric Hg by plant leaves, followed by litterfall, has been suggested to be important for understanding Hg sources in forest regions^34^. Published data on forest organic soils demonstrates significant negative δ^202^Hg values and a negative MIF, similar to the Hg isotopic compositions of the organic soils on Mt. Leigong^34^,^39^. A recent study showed that the decomposition of foliage does not generate significant changes in the Hg isotopic composition^34^. Indeed, the lush vegetation in the Mt. Leigong forest areas is likely to have sequestered Hg through complexation with organic matter. On Mt. Leigong, the surface soils generally contain moderately high organic matter (6.1 ± 3.9%, with a maximum of 18%) resulting from partially decayed plant matter. The organic matter contents were significantly correlated with both THg and MeHg in the topsoil (*r*^2^ = 0.16, *p* < 0.05 for THg; *r*^2^ = 0.51, *p* < 0.01 for MeHg; the *r*^2^ for THg can be increased to 0.41 if the outliers are excluded; Fig. S4) and this is presumably due to the well-documented strong affinity of terrestrial Hg for organic matter^3^,^17^,^18^. The bedrocks of Mt. Leigong generally have a negligible OM content, suggesting that the Hg associated with organic matter in the soil mostly originates from the decomposition of foliage^34^. On Mt. Leigong, the surface soils (δ^202^Hg: −2.63‰ to −1.42‰; Δ^199^Hg: −0.38‰ to −0.06‰) fall in between the bedrock (δ^202^Hg: −0.92‰ to −0.86‰; Δ^199^Hg: −0.04‰ to +0.01) and moss samples (δ^202^Hg: −2.37‰ to −2.09‰; Δ^199^Hg: −0.48‰ to −0.39‰) with respect to isotopic signature. Hg in the moss samples favourably corresponds to recent data on lichens^29^,^40^ and plant leaves^31^,^34^, and this may indicate the mixing of decaying foliage with geogenic Hg sources. Hence, the application of a simple MIF mass balance model to the soil system using the following equations is considered to be valid: 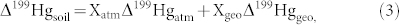

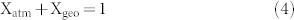


where the ‘atm’ and ‘geo’ subscripts refer to the atmospheric (i.e., litterfall) and geogenic sources, respectively. The Δ^199^Hg_geo_ is assumed to be zero given that MIF does not occur during the rock weathering process. According to the above-estimated isotopic compositions of the two end-members (the atmospheric and geogenic sources), the fraction of Hg that originated from the atmospheric source (X_atm_) and “the geogenic source (X_geo_)” can be evaluated using equations 3 and 4.

As indicated in Fig. 4, the isotope method demonstrated a very similar trend to that of the inert element method and revealed a significant atmospheric fractionation (>80%) at high elevations, with less than 40% in the foothills (*r*^2^ = 0.64–0.67, *p* < 0.01 for both). However, the inert element method revealed a higher atmospheric fraction at low elevations. These results confirm our hypothesis that there is an altitudinal magnification effect (i.e., mountain trapping effect) on atmospheric Hg inputs in montane soils, which may be indicative of the fate and transport of Hg at a regional or global scale.

Simple MDF (δ^202^Hg) binary mixing models have been successfully used to estimate Hg pollution sources in many ecosystems^40^,^41^. In the present study, the plot of δ^202^Hg and 1/THg displayed a positive linear correlation (*r^2^* = 0.48, *p* < 0.001) (Fig. S5), indicating the binary mixing of atmospheric Hg and geogenic Hg. A significant correlation (*r*^2^ = 0.68, *p* < 0.001) between δ^202^Hg and X_atm_ in the soil was observed (Fig. S6). Based on this correlation, the MDF signatures of the two end-members were obtained (Fig. S6). The δ^202^Hg from geogenic origin was approximately −1.38‰, and that from atmospheric deposition was approximately −2.50‰. The δ^202^Hg of the atmospheric Hg MDF compared to the observed δ^202^Hg in moss samples (δ^202^Hg of −2.21 ± 0.14‰, 2 s.d., n = 3) demonstrats that an MDF of −0.29 ± 0.14‰ may have occurred during the absorption of Hg by moss. Moss is an epiphytic plant that incorporates atmospheric Hg predominantly through the stoma. Isotope fractionations of heavy metals (e.g., Cu, Zn and Fe) have been demonstrated to occur, with a preferential translocation of light Hg isotopes to plants. Recent studies also demonstrated that plants (e.g., rice^31^ and lichen^36^) can preferentially incorporate light Hg isotopes during growth. The geogenic source of Hg is primarily derived from the weathering of bedrock. The type of bedrock in Mt. Leigong is uniform, and the average δ^202^Hg in the rock samples is −0.89 ± 0.10‰ (2 s.d., n = 2), which indicates that an MDF of −0.49 ± 0.10‰ in δ^202^Hg may occur during weathering. Mt. Leigong has a sub-tropical climate with an annual precipitation of 1250–1700 mm^11^. In such a wet climate, intense weathering may involve leaching. Recent leaching experiments of soils and Hg mine wastes clearly suggested that the more soluble Hg fractions are generally enriched with heavier Hg isotopes^42^. The δ^202^Hg of the atmospheric Hg (−2.50‰) is comparable with previous data on plants (e.g., rice^31^, aspen trees^34^ and lichen^36^), indicating that plants can preferentially incorporate light Hg isotopes during growth.

## Discussion

### Potential mechanisms for Hg isotope signatures in montane soils

Vertical variations of Hg isotopic composition in topsoils recorded in this study can be explained by isotope fractionation during Hg cycling in the forest ecosystem and/or mixing of Hg from different sources (e.g., atmospheric and geogenic origins). In mountain forest areas such as Mt. Leigong, the steep environmental gradients (e.g., temperature, precipitation and solar radiation) very likely influence the biogeochemical behaviour of Hg and lead to Hg isotope fractionation. The isotope fractionation of Hg in the Mt. Leigong elevation gradient may be a function of multiple physico-chemical processes, such as the evasion of Hg^0^ from soils, deposition of atmospheric Hg (e.g., precipitation, dry deposition and litterfall) and re-emission of wet-deposition Hg. To the best of our knowledge, the evasion of Hg from soils mainly involves processes such as photo-reduction^22^, volatilisation^24^,^43^ and the microbial reduction of soil Hg^37^. Generally, all these processes induce typical kinetic MDF values of Hg isotopes and produce Hg^0^ with significantly lower δ^202^Hg values than the original Hg^2+^. The photo-reduction of Hg may lead to the MIF of odd Hg isotopes^20^,^22^,^44^, whereas no significant MIF is recorded to be induced during volatilisation and microbial reduction processes^24^,^37^,^43^. A recent study by Demers et al^34^. also indicated that photo-reduction, volatilisation and microbial reduction could not be the major processes for the evaded Hg pool in forest areas. In soil humus such as that on Mt. Leigong, Hg binds strongly with thiols and other reduced sulphur groups associated with organic matter^9^. Soil evasion fluxes in pristine forest areas are generally extremely low because of the high organic matter content, suppression by leaf litter cover, and canopy shading^34^.

On Mt. Leigong, the surface soils have received Hg from geological sources (e.g., weathering) and atmospheric sources (dry and wet deposition). In this study, the Hg levels in two rock samples (0.10 ± 0.02 mg·kg^−1^, 2 s.d., n = 2) were lower than those in the soil samples. The δ^202^Hg (−0.89‰ ± 0.04, 2 s.d., n = 2) and the Δ^199^Hg (−0.02‰ ± 0.04, 2 s.d., n = 2) in the rock samples are consistent with the data from previous studies, as Hg in geogenic material (e.g., mineral deposits^45^, hydrothermal emissions^45^ and volcanoes^46^) generally have δ^202^Hg values of approximately −0.60‰, with no evidence of a significant MIF (Δ^199^Hg < 0.2‰)^19^.

Despite the proposed geogenic sources of Hg, atmospheric sources of Hg could also have been incorporated into the organic soils through wet (e.g., precipitation) and dry (e.g., particulate Hg and litterfall) atmospheric Hg deposition. Few studies have focused on the Hg isotope composition of precipitation and direct atmospheric Hg species^25^,^32^,^33^. It is worth noting that all of the described studies have demonstrated a significant MIF of even isotopes (e.g., ^200^Hg and ^204^Hg) in precipitation and direct atmospheric Hg samples^25^,^32^,^33^,^34^. Generally, Hg^0^ is characterised by negative Δ^200^Hg values, and precipitation (which contains mainly Hg^2+^ and Hg_p_) displays positive Δ^200^Hg values. In the present study, the absence of any MIF of even Hg isotopes in surface soils could be explained by the mixing of different atmospheric Hg species and precipitation. Alternatively, Chen et al^33^. suggested that the MIF of even Hg isotopes is likely linked to photo-initiated Hg^0^ oxidation, being controlled by stratosphere incursion, the presence of aerosols, oxidant intensity, solar irradiation and air mass movement. Several studies have reported near-zero Δ^200^Hg values in ambient gaseous Hg in the Great Lakes region^32^, the Arctic^25^ and near the Wanshan Mercury Mine^31^, indicating that photo-initiated Hg^0^ oxidation occurring in certain areas may not induce a significant MIF of even Hg isotopes. The mechanism for the MIF of even Hg isotopes is still unclear^33^, and further studies are needed.

In the present study, the significant negative MIF of odd Hg isotopes is established as an important feature of our investigated samples (i.e., lichens and surface soils). Two plausible mechanisms that might explain the odd-N MIF in this Hg isotope system include (1) the magnetic isotope effect (MIE)^47^ and (2) the nuclear volume effect (NVE)^48^. The Δ^199^Hg/Δ^201^Hg ratios of MIF produced by different mechanisms may be diagnostic. According to several recent studies, MIF occurring due to the NVE (e.g., Hg^0^ evaporation, abiotic dark reduction of Hg^2+^ and equilibrium Hg^2+^-thiol complexation) was estimated to result in a Δ^199^Hg/Δ^201^Hg ratio of 1.5 to 2.0^20^,^23^,^24^. The MIE has been documented during photochemical reactions of aqueous Hg species (e.g., MeHg and Hg^2+^). When Δ^199^Hg and Δ^201^Hg values are plotted for each of these photochemical processes, CH_3_Hg^+^ and Hg^2+^ photo-reduction have slopes of 1.36 and 1.00, respectively^22^. The sign of MIF produced by MIE is dependent upon the type of organic ligand involved^20^,^22^,^44^. As shown in Fig. 3, the negative Δ^199^Hg values of the surface soils from Mt. Leigong indicate a deficit of odd isotopes, which is in close agreement with the direct/indirect air samples and surface soils from other regions in the world^23^. The slope of approximately 0.98 that was obtained here for Δ^201^Hg/Δ^199^Hg (which is not compatible with the NVE) indicates that a portion of Hg in the soil samples in this region may have undergone photo-reduction processes before being stored in continental pristine soils.

### Potential mechanisms for Hg magnification in montane soils

**Figure 5:**
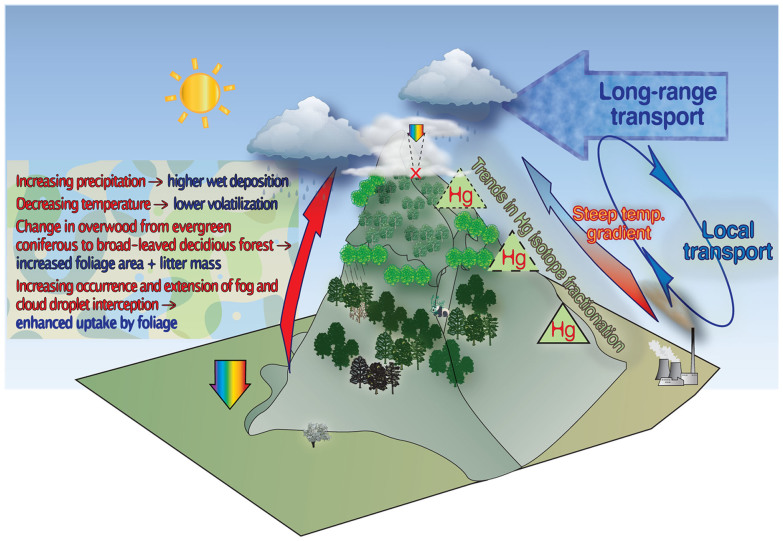
Illustration of potential mechanisms for mercury magnification in montane soils (by Hua Zhang and Jonas Sommar).

Whether the levels of Hg, a typical volatile pollutant, are increased in the montane soils at higher elevations in colder zones depends primarily on the distinction of the relationship between the ‘source’ and the ‘sink’ of Hg and the corresponding geochemical processes that are influenced by the elevation difference. A suite of controls that may cause the preferential accumulation of Hg at higher altitudes in the investigated montane area are depicted in Fig. 5 and are addressed in the following discussion.

(1). Litterfall. Litterfall is a critical Hg input to mountain forest ecosystems in autumn, when deciduous trees enter dormancy and their leaves senesce^17^. Hg^0^ flux measurements over deciduous forest ecosystems have indicated growing seasonal patterns from significant net deposition following leafing to net emission towards the end of the foliar season^49^,^50^. This finding primarily reflects the assimilation of Hg^0^ by the foliage via the stomata and cuticle over time. Furthermore, Hg tends to become enriched in forest litter compared with aboveground fresh foliage (50%–800%)^17^,^18^. Dry deposition (litterfall) can account for 40% and 80% of the total Hg mass entrainment in the forest soil in winter and spring, respectively^17^,^18^. In a previous communication^10^, we reported that Hg depositional fluxes in the summit zone of Mt. Leigong were, on a yearly basis, substantially dominated by litterfall with minor contributions from precipitation and throughfall (39.5, 6.1 and 10.5 μg.m^−2^.yr^−1^, respectively).

Moreover, the foliage/air partition coefficient increases with lower temperatures and higher elevations^51^ (i.e., plant leaves may retain more atmospheric Hg at higher elevations). In a broad survey of background US forests, Obrist et al. identified latitude, in addition to factors such as precipitation, as a suitable predictor of the Hg burden in litter^17^. The foliar uptake of atmospheric Hg mediated by intercepting cloud/fog water may also constitute a viable pathway for Hg accumulation. Although a firm conclusion is inhibited by the scarcity of observations, the Hg concentration in clouds and fogs has been observed to be elevated compared with that in precipitation^8^,^52^, especially in persisting and stationary fog^52^. The positive correlation (*r*^2^ = 0.33, *p* < 0.01; Fig. S3) between the Hg concentrations in leaf litter samples and elevation may reflect increased foliage uptake promoted by lower temperatures and extensive cloud water contact at higher elevations.

In the canopy flora of Mt. Leigong, there is a transition (1400–1800 m, evergreen coniferous species mixed with broad-leaved deciduous) from a domination of evergreen coniferous species in the foothills (<1400 m) to broad-leaved deciduous forests in the higher elevation zones (>1800 m)^11^,^12^ (Fig. 5), which suggests an increase in areal litterfall mass at higher elevations. Therefore, the higher litterfall mass combined with the higher Hg concentrations in the leaf litter at high elevations described above further suggest that litterfall decomposition may play an important role in the amplification of soil Hg on Mt. Leigong.

(2). Temperature. A certain proportion of the Hg deposited into the forests is in labile forms that can be re-emitted (as Hg^0^ after reduction) in direct competition to the process of incorporation with the soil matrix through complexation^18^. Hg^0^ emission fluxes from terrestrial surfaces are influenced by the substrate (e.g., soil) temperature^7^,^17^,^18^,^53^, with high temperatures facilitating Hg^0^ volatilisation^53^,^54^. In addition, the aqueous (photo-)reduction of Hg^2+^ to Hg^0^ is facilitated in warmer vegetation zones exposed to more abundant sunlight^55^. Hence, decreasing temperature at high elevations^16^ supports a suppression of the Hg^0^ air–surface exchange, thereby indirectly enhancing the retention of Hg in the soil compartment.

One process affecting certain persistent organic pollutants, termed the ‘grasshopper effect’, may also be engaged in the Hg enrichment process in montane soils. In this process, Hg evaporates from warmer zones in neighbouring lowlands (especially where pollution sources exist), travels through the atmosphere and is deposited in cooler, higher montane zones when the temperature drops. This process can be repeated in ‘hops’ (Fig. 5). However, the repeated mountain ‘hops’ (local transport) may be relatively limited and might insignificantly contribute to Hg enrichment in remote montane soils compared with long-range transport processes^1^,^53^. Additionally, a diurnal variation of wind patterns controlled by temperature has previously been proposed^51^ as an important driver of volatile pollutants in mountain regions. Specifically, more volatile contaminants are carried by upslope winds in warmer daytime temperatures than by downslope winds in cooler night-time temperatures.

(3) Precipitation. Orographic effects driven by temperature at higher elevations would result in greater Hg wet deposition due to higher precipitation relative to neighbouring lowlands^8^. Atmospheric hydrometeors (rain drops and snow) are very efficient scavengers of aerosol particles and ionic Hg species^8^. In the context of quantifying atmospheric wet deposition processes, the ratio of a chemical's concentration in precipitation to its concentration in the air is known as the scavenging ratio, *W*. The *W* values reported in the literature for Hg-p span a large range from 300 to 1500^56^. Compared with Hg^0^ and Hg-p, GOM exhibits greater dry deposition velocities^57^. Even without experimental evidence, the *W* value of GOM applied in models is usually treated as that of an acidic gas (e.g., HNO_3_). Simple theoretical considerations have indicated that *W* is a function of inverse temperature. Drevnick et al. (2010)^13^ reported a significant increase in *W* with increasing altitude in the western U.S., which is consistent with our recent observations in southwestern China^58^. A recent study by Huang and Gustin (2012)^7^ also indicated higher levels of Hg wet deposition at sites with higher elevations.

On Mt. Leigong, precipitation increases with the altitudinal decrease of temperature (detailed in the Supporting Information)^11^,^12^. Significantly positive correlations between the soil Hg levels and precipitation or the inverse of temperature (*r*^2^ = 0.67–0.69, *p* < 0.01 for both) (Fig. S7) were observed in the present study, and these relationships may be indicative of the enhanced retention/deposition of Hg in high-elevation soils due to the temperature- and precipitation-related mechanisms described above. However, other mechanisms may also contribute to the altitudinal enrichment processes. For example, solar radiation has been suggested as a significant factor controlling the Hg flux between the soil and atmosphere^59^. Solar radiation decays with rising elevation on Mt. Leigong^26^ and in most alpine regions (due to increased cloud cover and an increased number of rainy days)^16^, thereby limiting direct photolytic degradation^1^, which, in turn, reduces the Hg emissions from the land surface to the atmosphere.

On Mt. Leigong, the temperature and solar radiation decrease and the precipitation, fog/cloud and air humidity increase with increasing elevation^11^,^12^,^26^. Hence, re-emission (the ‘grasshopper effect’) produces a negative influence on the sequestration of atmospheric Hg and decreases the Hg concentration, especially at low elevations. In contrast, scavenging of atmospheric Hg by precipitation and enhanced litterfall provoke increases in the soil Hg concentration at higher altitudes. These processes may be the main reasons that explain the increased Hg concentrations in soil samples with rising elevation.

### Implications for regional or global Hg cycling

A negative elevational dependence was observed in the MDF and MIF signatures of Hg isotopes. The application of a MIF (Δ^199^Hg) binary mixing approach and the traditional inert element method unanimously indicated that the fraction of Hg derived from the atmosphere distinctly increased with altitude. Our study, for the first time, demonstrates that a ‘mountain trapping effect’ of semi-volatile Hg can occurs in montane environments and provides a systematic discussion of the possible mechanisms. Mercury magnification in high-elevation montane soils is likely driven by the altitudinal dependence of temperature, precipitation, litterfall and other factors (e.g., solar radiation), Of these factors, litterfall may be the most critical. Our observations infer that previous studies on regional or global Hg cycles/distribution may have significantly underestimated the Hg mass trapped by mountainous regions, as mountains account for a significant proportion of the global terrestrial area. Our study shows that Hg stable isotope ratios can be used to track atmospheric Hg deposition in upland forest systems. This technique may be useful in future studies that assess environmental changes in montane forest ecosystems.

## Methods

The THg concentrations were measured in the soil and rock samples using cold-vapour atomic absorption spectrometry (CVAAS), and the THg concentrations in the moss and leaf litter samples were determined using the dual-stage gold amalgamation method and cold-vapour atomic fluorescence spectrometry (CVAFS) detection following USEPA method 1631. The soil MeHg concentrations were determined using aqueous ethylation, purge, trap and GC CVAFS detection following USEPA method 1630. The Hg isotopic ratios were determined with MC-ICP-MS using a Nu-Plasma mass spectrometer equipped with 12 Faraday cups. The quality-control system for the Hg concentration analyses consisted of method blanks, blank spikes, matrix spikes, certified reference materials and blind duplicates. The reproducibility of the isotopic data was assessed after measuring replicate sample digests (typically n = 2). We also analysed the UM-Almadén as a secondary standard (once every 10 samples) in addition to the bracketing standard NIST 3133. The Hg concentration in the UM-Almadén was measured with the same method as other samples in each analytical session.

Detailed information regarding the site description, sample collection and preparation, analysis methods for determining the Hg species concentration and the Hg isotope ratio, quality assurance and control, and climatic parameter estimation methods is provided in the Supplementary Information.

## Author Contributions

H.Z., R.S.Y., X.B.F. and T.L. conceived the project. H.Z., R.S.Y., X.W.F. and A.S. organised the sampling. H.Z. and R.S.Y. measured the Hg species concentrations and the Hg isotope ratios. H.Z., R.S.Y., X.B.F., T.L., C.W.N.A. and J.S. analysed and interpreted the data. H.Z. and R.S.Y. wrote the paper with comments from all authors.

## Supplementary Material

Supplementary Information Supplemental Material
