# Supplementary material for: Atmospheric mercury inputs in montane soils increase with elevation: evidence from mercury isotope signatures
Source: Sci Rep. 2013 Nov 25;3:3322. doi: 10.1038/srep03322 (PMC3839031; doi:10.1038/srep03322)
Supplement: Supplementary Information — Supplemental Material [file srep03322-s1.doc]

***Supplemental Material for***

**Atmospheric mercury inputs in montane soils increase with elevation: evidence from mercury isotope signatures**

Hua Zhang,^1, 2, †^ Run-sheng Yin,^3, †^ Xin-bin Feng,^1, *^ Jonas Sommar,^1^ Christopher W. N. Anderson,^4^ Atindra Sapkota,^1^ Xue-wu Fu,^1^ and Thorjørn Larssen^2^

^1^ State Key Laboratory of Environmental Geochemistry, Institute of Geochemistry, Chinese Academy of Sciences, Guiyang, 550002, China

^2^ Norwegian Institute for Water Research (NIVA), Gaustadalléen 21, 0349 Oslo, Norway

^3^ State Key Laboratory of Ore Deposit Geochemistry, Institute of Geochemistry, Chinese Academy of Sciences, Guiyang, 550002, China

^4^ Soil and Earth Sciences Group, Institute of Agriculture and Environment, Massey University, Palmerston North, New Zealand

^*^Corresponding author:

Xin-bin Feng, phone: 86-851-5891356; fax: 86-851-5891609; e-mail: [fengxinbin@vip.skleg.cn](mailto:fengxinbin@vip.skleg.cn).

^†^These authors contributed equally to this work.

S1. Sample collection and preparation.

S2. Hg concentrations analyses.

S3. Hg isotope analyses.

S4. Climatic parameters estimation methods.

14 pages (including title page)

7 Figures (S1- S7)

**S1. Sample collection and preparation.**

Two sampling campaigns were conducted. In September 2009, soil, moss (from tree trunks) and litterfall samples were taken from 21 sites between elevations of 823 m a.s.l. and 2,178 m a.s.l at approximately 50 m intervals. In September 2010, 28 soil samples and 15 moss samples (from tree trunks) were collected from the same route (moss samples were only collected at >1,400 m as we worked too late on the mountain). All of the soil samples in both samplings were collected from the top layer, at depths of 0-5 cm. Each composite sample from the site consisted of five subsamples (quincunx sampling pattern). Two bed-rock samples from fresh outcrops were also collected at elevations of 1413 m and 2178 m a. s. l. All of the samples were individually sealed in polyethylene bags. Fresh green tissue was removed from the moss samples with scissors for laboratory analyses. The moss and leaf litter samples were washed with MilliQ water for approximately 30 minutes using an ultrasonic bath treatment (Ultrasons-H, Selecta). The samples were dried at 40 °C until a constant weight was obtained and later ground separately using a portable handheld grinder. The grinder was vigorously rinsed with ultrapure water and dried with a hair drier three to five times before each sample cycle. Soil and rock samples were dried, ground, homogenized, and later put through a sieve of 200 mesh prior to digestion.

**S2. Hg concentrations analyses.**

The Hg analysis methods have been detailed in our recently published paper ^4^. Briefly, for THg analyses, the ground soil and rock samples were digested in a water bath (95 °C) using a fresh mixture of HCl (12 mol · L^-1^) and HNO_3_ (16 mol · L^-1^) (1:3, v/v), and the moss and leaf litter samples were digested with a mixture of HNO_3_ (16 mol · L^-1^) and H_2_SO_4_ (18.4 mol · L^-1^) (4:1, v/v). The THg levels were measured in the soil and rock samples using cold-vapour atomic absorption spectrometry (CVAAS), and the THg concentrations in the moss samples and leaf litter samples were determined using the dual-stage gold amalgamation method and cold-vapour atomic fluorescence spectrometry (CVAFS) detection, following USEPA method 1631. MeHg analyses of the soil samples were prepared using the CuSO_4_-methanol/solvent extraction method and determined using aqueous ethylation, purge, trap, and GC CVAFS detection, following USEPA method 1630. Similarly, the titanium (Ti) in the soil sample was analysed using the Axios (PW4400) X-ray Fluorescence Spectrometry system (PANalytical, B.V. Netherlands). Approximately 5 g of powdered and homogenised soil samples were mixed with approximately 1 g of boric acid (H_3_BO_3_) and pressed to form pellets. The detection limit for Ti was in the range of 1 mg per kg. The soil pH was measured using a pH electrode, with a solid:water ratio of 1:2.5 ^5^. The organic matter (OM) contents of the soil samples were determined using the potassium dichromate volumetric method coupled with water heating ^6^.

The quality control system for Hg analyses consisted of method blanks, blank spikes, matrix spikes, certified reference materials and blind duplicates. All of the method blanks were below the corresponding limits of detection (0.01 µg.kg^-1^ (CVAFS) THg in the moss and leaf litter, 0.01 mg.kg^-1^ (CVAAS) THg in the soil samples and 0.03 µg.kg^-1^ (CVAFS) MeHg in soil samples). For the moss and leaf litter sample measurements, a mean THg concentration of 5.4 ± 0.5 µg.kg^-1^ was obtained from GBW10010 (certified value of 5.3 ± 0.5 µg.kg^-1^). For the soil sample measurements, a mean THg concentration of 0.30 ± 0.03 mg.kg^-1^ was obtained from GBW07405 (certified value of 0.29± 0.04 mg.kg^-1^), and a MeHg concentration of 5.61± 0.56 µg.kg^-1^ was obtained from IAEA 405 (certified value of 5.49 ± 0.53 µg.kg^-1^). The percentage of MeHg recovered from the spiked soil samples ranged from 83% to 117%. The relative percentage difference between the duplicate samples was < 8% for THg and MeHg.

**S3. Hg isotope analyses.**

The Hg isotopic ratios were determined with MC-ICP-MS using a Nu-Plasma mass spectrometer equipped with 12 Faraday cups ((Nu Instruments, Great Britain)) at the State Key Laboratory of Environmental Geochemistry, Institute of Geochemistry, Chinese Academy of Sciences, Guiyang, China. The sample introduction system consisted of a continuous flow cold-vapour generation system (CV) (HGX-200, CETAC U.S.) coupled to an Apex-Q desolvation unit (Elemental Scientific Inc., U.S.) for Hg and Tl introduction, respectively. SnCl_2_ was used as reducing agent and mixed according to Hg standards or sample digests to generate volatile elemental Hg. The Hg^0^ vapour from the CV generation system was mixed with a dry Tl aerosol produced via the desolvation device. Instrumental mass bias correction was achieved using Tl as an internal standard (20 μg L^-1^, NIST SRM 997) and external standard-sample bracketing with a NIST SRM 3133 Hg solution. An exponential fractionation law was applied for internal mass bias correction assuming a reference value of 2.38714 for the ratio ^205^Tl/^203^Tl. Data were acquired by monitoring ^198^Hg, ^199^Hg, ^200^Hg, ^201^Hg, ^202^Hg, ^203^Tl and ^205^Tl isotopes for a period of 10 min (1 block, 100 cycles with 6 sec integrations). A typical sequence consisted of measuring the NIST SRM 3133 Hg solution before and after each sample. To ensure optimum results of instrumental mass bias correction, the concentration of the bracketing solution was systematically adjusted to within 10% of the Hg concentration in the sample digest (typically 5 μg/L). Instrument blanks were analysed after each sample and each bracketing standard and on-line subtracted. Typical blank values were 10 mV for ^202^Hg and 30 mV for ^205^Tl, insignificant relative to typical sample and standard signals of 1 V for ^202^Hg and 3 V for ^205^Tl. A more detailed description of the overall instrumental setup, the parameters and the analytical conditions used throughout this study was presented in a previous paper ^7^.

Here, the Hg isotopic variations are reported in delta notation in units of per mille (‰) and referenced to the NIST SRM 3133 Hg standard (analysed before and after each sample), indicated here:

$\delta^{xxx}Hg(^{0}/_{00})=\left\{[\frac{^{xxx}Hg/^{198}Hg_{sample}}{^{xxx}Hg/^{198}Hg_{NISTSRM3133}}]-1\right\}\times1000$, (equation 1)

Where xxx is the mass of each Hg isotope between 199 and 202 amu. The MIF is reported in the “capital delta” notation (Δ^xxx^Hg, deviation from mass dependency in units of per mille, ‰) and is the difference between the measured δ^xxx^Hg and the theoretically predicted δ^xxx^Hg value using the following formulas ^8^:

Δ^201^Hg ≈ δ^201^Hg － (δ^202^Hg * 0.7520), (equation 2)

Δ^200^Hg ≈ δ^200^Hg － (δ^202^Hg * 0.5024), (equation 3)

Δ^199^Hg ≈ δ^199^Hg － (δ^202^Hg * 0.2520). (equation 4)

The reproducibility of the isotopic data was assessed after measuring replicate sample digests (typically, n = 2). We also analysed the UM-Almadén as a secondary standard (once every 10 samples) in addition to the bracketing standard NIST 3133. The Hg concentration in the UM-Almadén was measured using the same methods that were used for the other samples in each analytical session. The overall average and uncertainty of δ values for all UM-Almadén measurements (2SD, n=15) agree well with the data reported by Blum and Bergquist (2007) ^9^. The uncertainties reported in this paper correspond to the larger value of either the measurement uncertainty of replicate sample digests or the uncertainty of the repeated measurements of the same digest during different analysis sessions. When the calculated 2SD was smaller than the replicate analyses of the reference UM-Almadén material, the uncertainty associated with UM-Almadén was used.

**S4.** **Climatic parameters estimation methods.**

The climatic parameters of the sampling site at different heights (H) on the mountain could not be directly measured in the field during the sampling period. Therefore, the average temperature (T) was estimated from verified monitoring data with a lapse rate of 0.46 °C per 100 m (T=19.21-0.46×H) ^1,3^. The average annual precipitation (P) on the south-west slope was estimated with an increased rate of 28.9 mm/100 m (P=972.6+28.9×H or P=1187+0.92×H^2^) ^2^. These equations were derived from three years of continuous precipitation monitoring data that were gathered by the Guizhou Climatic Data Centre, China, from 21 weather stations at different heights of the Mt*.* Leigong area. The differences between the predicted values and the monitoring data were only ±(0.1-11.4) mm ^2^.

**Figure S1. Location of *Mt.* Leigong in Guizhou Province, China (modified from reference 10, which is distributed under the Creative Commons Attribution 3.0 License).**

**Figure S2. THg concentrations by elevations in the moss samples.**

**
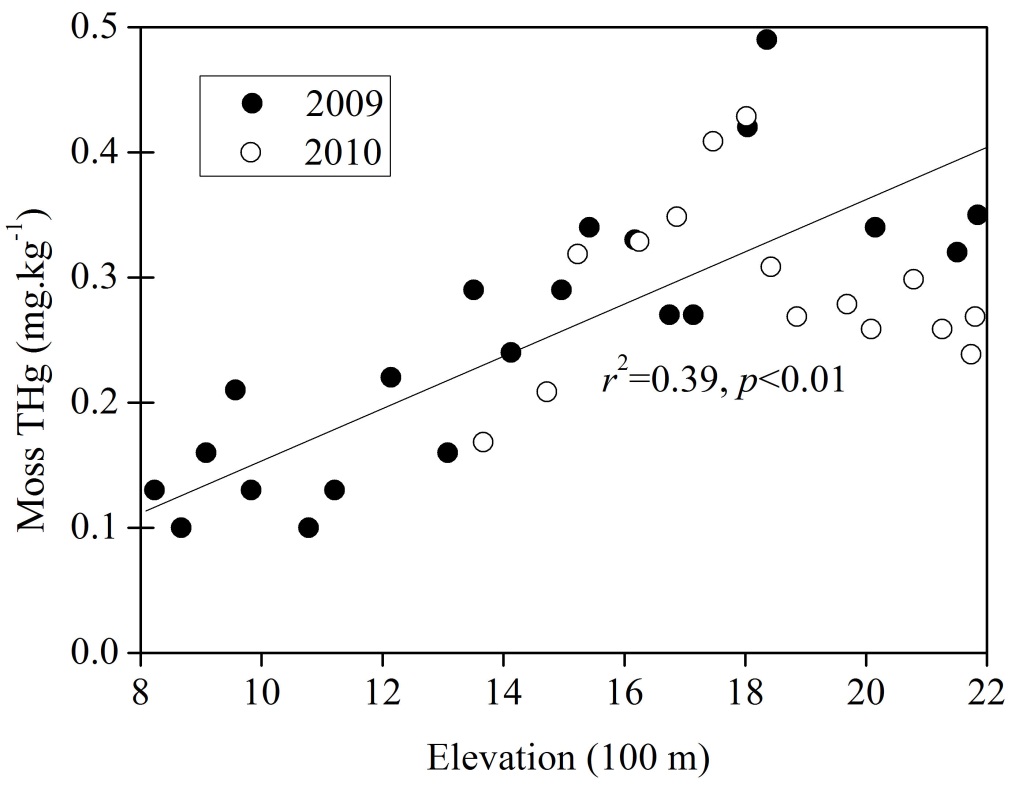
**

**Figure S3. THg concentrations by elevations in the litterfall samples.**


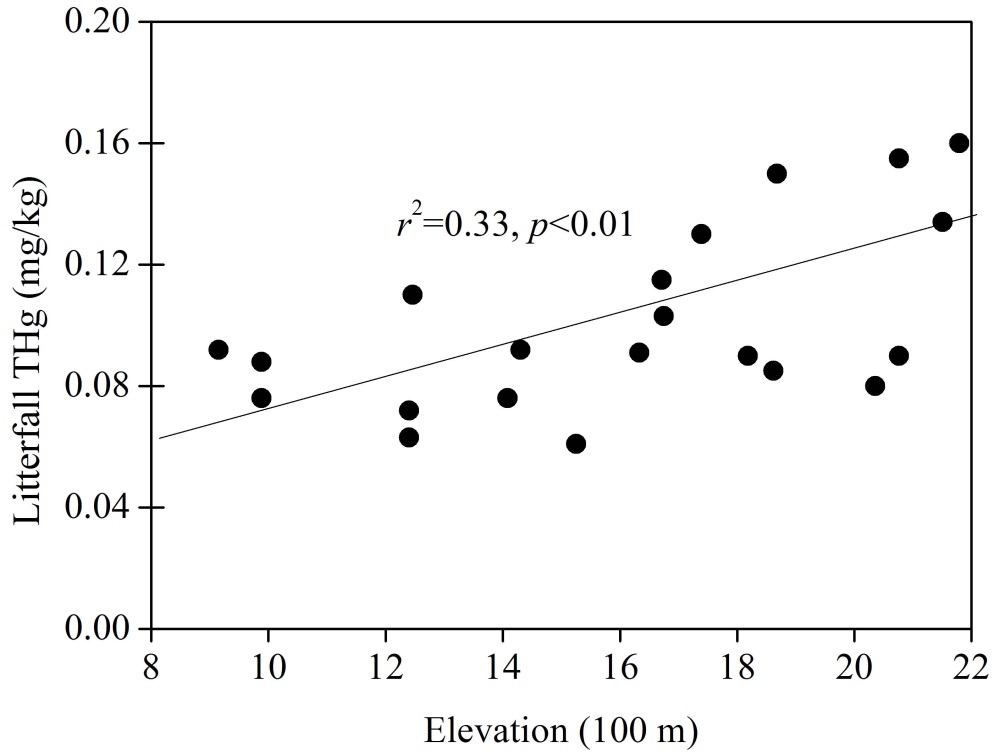


**Figure S4. Hg concentrations by organic matter content in the soil samples.**

**
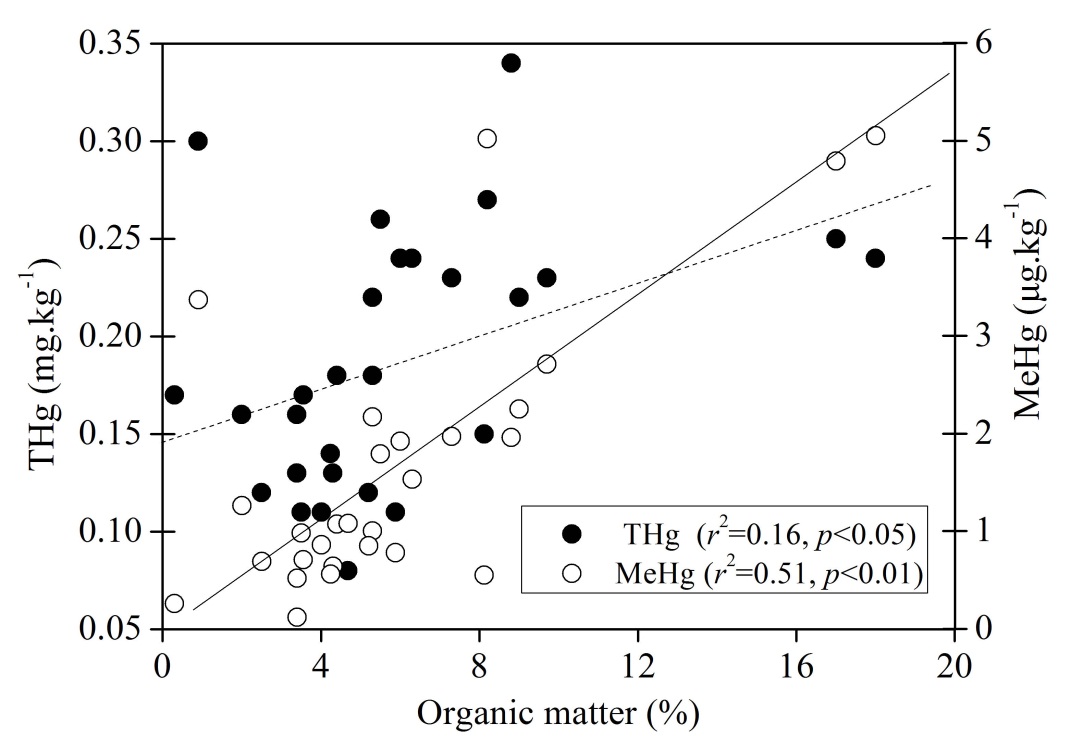
**

**Figure S5. The relationship of 1/THg to Δ^199^Hg, δ^202^Hg in the soil samples.**

**Figure S6. The relationship of δ^202^Hg to *X_atm._* (fraction of atmospheric Hg) in the soil samples .**

**Figure S7. Hg concentrations in soil samples by precipitation and 1/temperature.**

**
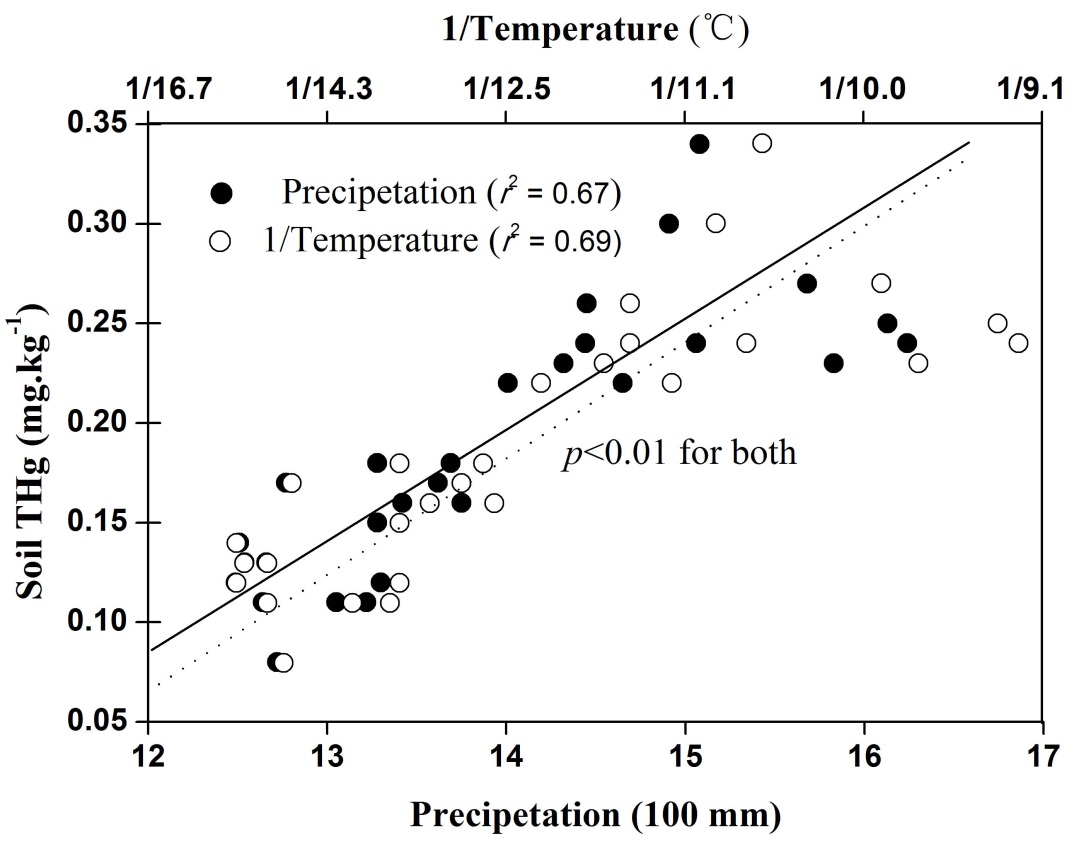
**

**References**

1 Yang, S. L. Protection and Rational Utilization of Natural Resources from the vertical natural zones in Mt. Leigong. *Journal of Guizhou Normal University (Natural Sciences)* **2**, 34-38 (1990).

2 Jin, H. T. Distribution Characteristics of Precipitation in Mt. Leigong. *J. Guizhou Meteorol.* **2**, 34-38 (1990).

3 Xiong, Y. H., Yang, S. Z. & Yang, J. Temperature trend in recent 46a years in Mt. Leigong. *J. Guizhou Meteorol.* **32**, 21-23 ( 2008).

4 Zhang, H. *et al.* Selenium in Soil Inhibits Mercury Uptake and Translocation in Rice (Oryza sativa L.). *Environ. Sci. Technol.*, doi:10.1021/es302245r (2012).

5 MAC. Soil Testing Part 2: Method for Determination of Soil pH; Ministry of Agriculture of the People’s Republic of China: Beijing. (2006).

6 Lu, R. Chemical Analysis Method of Agricultural Soil (in Chinese), China Agricultural Science Press, Beijing, pp: 106–107. (2000).

7 Yin, R. *et al.* High Precision Determination of Mercury Isotope Ratios Using Online Mercury Vapor Generation System Coupled with Multi-collector Inductively Coupled Plasma-Mass Spectrometer. *Chinese J. of Anal. Chem.* **38**, 929–934 (2010).

8 Smith, C. N., Kesler, S. E., Klaue, B. & Blum, J. D. Mercury isotope fractionation in fossil hydrothermal systems. *Geology* **33**, 825-828 (2005).

9 Blum, J. D. & Bergquist, B. A. Reporting of variations in the natural isotopic composition of mercury. *Anal. Bioanal. Chem.* **388**, 353-359 (2007).

10 Fu, X. W. *et al.* Atmospheric gaseous elemental mercury (GEM) concentrations and mercury depositions at a high-altitude mountain peak in south China. *Atmos. Chem. Phys.* **10**, 2425-2437 (2010).
